# The Effect of Ultraviolet B Irradiation Compared with Oral Vitamin D Supplementation on the Well-being of Nursing Home Residents with Dementia: A Randomized Controlled Trial

**DOI:** 10.3390/ijerph17051684

**Published:** 2020-03-05

**Authors:** Bistra I. Veleva, Monique A. A. Caljouw, Jenny T. van der Steen, Bart J. A. Mertens, Victor G. M. Chel, Mattijs E. Numans

**Affiliations:** 1 Department of Public Health and Primary Care, Leiden University Medical Center, P.O. Box 9600, 2300 RC Leiden, The Netherlands; M.A.A.Caljouw@lumc.nl (M.A.A.C.); J.T.van_der_Steen@lumc.nl (J.T.v.d.S.); V.G.M.Chel@lumc.nl (V.G.M.C.); M.E.Numans@lumc.nl (M.E.N.); 2 Woonzorgcentra Haaglanden, Polanenhof 497, 2548 MP Den Haag, The Netherlands; 3 Department of Medical Statistics and Bioinformatics, Leiden University Medical Center, P.O. Box 9600, 2300 RC Leiden, The Netherlands; B.J.A.Mertens@lumc.nl

**Keywords:** UVB irradiation, vitamin D supplementation, well-being, quality of life, nursing home residents

## Abstract

There are indications that ultraviolet B (UVB) exposure has beneficial effects on well-being through mechanisms other than vitamin D synthesis alone. We conducted a randomized controlled multicenter trial to compare the effects of UVB light and vitamin D supplementation (VD) in terms of the well-being of nursing home residents with dementia. Participants were randomly assigned to the intervention group (UVB group, *n* = 41; half-body UVB irradiation, twice weekly over 6 months, with 1 standard erythema dose (SED)) or to the control group (VD group, *n* = 37; 5600 International units (IU) cholecalciferol supplementation once a week). The main outcome was well-being, measured by the Cohen-Mansfield Agitation Inventory (CMAI) and the Cornell scale for depression in dementia at 0, 3, and 6 months. Secondary outcomes were QUALIDEM quality of life domains and biochemical parameters of bone homeostasis. Intention-to-treat analysis with linear mixed modeling showed no significant between-group differences on agitation (*p* = 0.431) or depressive symptoms (*p* = 0.982). At six months, the UVB group showed less restless/tense behavior compared to the VD group (mean difference of the mean change scores 2.2, 95% CI 0.8 to 3.6; *p* = 0.003 for group x time interaction) and lower serum 25(OH)D3 concentration (estimated mean difference - 21.9, 95% CI −32.6 to −11.2; *p* = 0.003 for group difference). The exposure of nursing home residents with dementia to UVB light showed no positive benefits in terms of wellbeing. UVB treatment may have a positive effect on the restless/tense behavior characteristic of advanced dementia but more research is needed to confirm this finding.

## 1. Introduction

Observational studies indicate that low sun exposure increases the risk of cardiovascular disease [1,2] and that there is a strong inverse relationship between all-cause mortality and sun exposure [1]. Therapy using ultraviolet (UV) light is an important treatment option for several skin diseases [3]. A mood-enhancing effect of UV light has also been reported [4,5,6,7,8,9]. UV light acting on the skin is absorbed by 7-dehydrocholesterol in the plasma membrane of epidermal cells, resulting in production of previtamin D3 [10], the major source (90%–95%) of vitamin D for most vertebrates, including humans [2].

Inadequate sun exposure leads to vitamin D deficiency and insufficiency [11,12]. Supplementation of vitamin D in old age is an important field of study in geriatrics. The nursing home population is at particular risk of sun-deprivation because of disease and disability, the limited resources and staffing in nursing homes, and a lack of organizational modalities [13,14,15]. A pilot trial of an eight-week course of weekly, frontal half body irradiation with ultraviolet B (UVB) of nursing home residents in a Dutch nursing home showed a significant increase in 25(OH)D3 [11].

Induction of cutaneous vitamin D production by using UVB exposure may be preferable to oral supplementation amongst older nursing home residents because it cannot induce toxic levels, it helps prevent polypharmacy and it is plausible that vitamin D synthesis is not the sole mechanism by which sunlight or UVB light exerts its beneficial effects on human health and well-being. Getting older is inevitably accompanied by perceiving a continuous loss in functioning, healthy state and social engagements, and this process is strongly delineated and progressive in persons with dementia [16]. Improving wellbeing (feeling of happiness, sadness, stress and pain) empowers adaption abilities [17], and this can be especially meaningful in the population with advanced dementia where agitation is a persistent and most common symptom and often requires intensive pharmacological management [18].

Therefore, the aim of this study is to compare the effect of UVB irradiation to oral vitamin D supplementation on well-being in nursing home residents with advanced dementia.

## 2. Materials and Methods

This study had a randomized controlled multicenter trial design. Written informed consent was obtained from the legal representatives of all participants. The study was conducted in accordance with the declaration of Helsinki, and protocol was approved by the Medical Ethical Committee of Leiden University Medical Center (Registration No P16.010) on 11 April, 2016 and was registered in the Dutch Trial Register (NL5704).

Participants were recruited from three nursing homes connected to the University Network for the Care sector South Holland (UNC-ZH). Team leaders of the nursing homes sent information letters with an informed consent form to all nursing home residents and their families. An independent physician with a specialty in internal medicine was assigned to answer the questions of the participants and their families.

Inclusion criteria were a diagnosis of dementia and an age exceeding 70 years, while exclusion criteria were (1) actinic keratosis, (2) skin cancer, (3) porphyria, (4) sun allergy, (5) use of drugs that may induce photodermatosis, (6) hypercalcemia, (7) use of vitamin D fortified food, (8) anxiety, agitation or resistance to bodily contact. Examination of the participants for actinic and cancer skin lesions, as well as skin type according to the Fitzpatrick scale [19], was performed by a dermatologist.

The participants were randomized in blocks of four and assigned to either receive the intervention (UVB light; UVB group) or standard vitamin D treatment (control; VD group). The group assignment files were placed in sequentially numbered opaque sealed envelopes to conceal the sequence until individual interventions were assigned. Intervention delivery and outcome assessment was not blinded. Nursing staff administered the medication, intervention and questionnaires to avoid disturbance of the daily routine.

The sample size was calculated on the basis of the Cohen-Mansfield Agitation Inventory (CMAI), an instrument measuring agitation and covering 29 behavioral items, each rated on a 7-point Likert scale of frequency (varying from never to several times an hour). Summed scores ranged from 29 to 203. Assuming a standard deviation (SD) of 13 points, an ά of 0.05 and an estimated drop-out of 40%, a sample of 80 patients would provide an 80% probability of detecting a mean between group differences of 10 points.

The intervention consisted of half body UVB-irradiation with 1 standard erythema dose (SED). The procedure was carried out twice a week with a portable, tilting sunbed canopy (Topaz 10 V, VDL Hapro Laboratory, Kapelle, the Netherlands) positioned at a fixed distance of 75 cm above a bed. The standard tanning lamps were replaced with UVB spectrum lamps (F71T12 100W Preheat-Bipin, Cosmedico, Stuttgart, Germany). Lamp light emission consisted of UVB—5.013 W·m^−2^, ultraviolet A (UVA)—4.650 W·m^−2^, ultraviolet C (UVC)—0.00001W·m^−2^, with UVB accounting for 54.6% of the spectrum. The exposure time was set at eight minutes a session, which was safeguarded by an electronic timer to prevent unintended longer exposure. Protective glasses were worn during treatment. The total treatment time over 6 months was 432 min. UVB treatment was discontinued when participants clearly objected or showed signs of discomfort on two consecutive sessions. They were then removed from the UVB exposure group and started again on vitamin D capsules. The control group received vitamin D capsules, 5600 IU cholecalciferol supplementation once a week, which is the standard treatment dose for all older persons >70 years according to the Dutch Health Council [20]. All nursing home residents in the Netherlands receive this standard supplementation. In the UVB group, vitamin D supplementation was stopped one week after drawing blood for the baseline biochemical parameters and one week before starting the intervention.

The primary outcome was well-being, monitored with the CMAI and the Cornell scale for depression in dementia at 0, 3 and 6 months. Higher CMAI scores indicate a more frequent display of agitated behavior [21,22]. The Cornell scale for depression in dementia was used to assess mood. It consists of 19 questions classified in 5 categories: mood-related signs, behavioral disturbance, physical signs, cyclic functions and ideational disturbances. Scores higher than twelve indicate probable major depression [23]. Secondary outcome measures were quality of life (QoL), serum 25(OH)D3 concentration and biochemical parameters of bone homeostasis. The QUALIDEM (shortened version) was used to assess QoL [24] and consists of 18 items covering 6 domains of QoL, including care relationship, positive affect, negative affect, restless tense behavior, social relations and social isolation. The higher the score on a subscale, the better the person does on this particular QoL domain. Serum levels of 25(OH)D3 were measured using an electrochemiluminescence immunoassay (ECLSA, Roche diagnostics, Basel, Switzerland). Serum creatinine, parathyroid hormone (PTH), calcium and phosphate were measured at 0 and 6 months. The assessments were performed by the nursing staff, and at least two experienced nurses discussed them and completed the forms. Bone homeostasis parameters were measured at the biochemical laboratory of Leiden University Medical Center.

Information on participant’s sociodemographic characteristics (gender, age and skin type) and dementia severity were obtained at the baseline. Dementia severity was assessed using the Bedford Alzheimer Nursing Severity Scale (BANS-S), which is composed of 7 items, scaled 7–28, and a score of 17 or higher indicates severe dementia.

Statistical analyses were performed in SPSS 23.0 (IBM Corp. Released 2015, Armonk, NY, USA) in accordance with the intention-to-treat principle. Descriptive statistics were used to outline the basic characteristics of the study population. The results are reported using the mean and standard deviation (SD) for normally distributed variables and median and interquartile range (IQR) for non-normally distributed variables. Pearson’s chi-square test, student’s t-test and Mann-Whitney U-tests were used to test differences between the baseline measurements in the intervention and control groups. A *p*-value <0.05 was considered statistically significant. Analysis of treatment effects was conducted using linear mixed models that accounted for repeated measurements in the subjects, estimated using restricted maximum likelihood (Brady T.West, 2009) [25]. Time was treated as a categorical variable. As fixed effects, we entered randomization, time, randomization-by-time interaction and the baseline of the outcome measure. Visual inspection of residual plots did not reveal any obvious deviations from homoscedasticity or normality. The following effects were estimated for the outcome variable: the main effect of the intervention, the main effect of time (at three time points) and the interaction between group and time. The treatment effect was presented at each time point as an estimated difference between the mean change score per group (95% CI) with the VD group as a reference. If the missing items on the Cornell depression scale were up to five, they were imputed as a mean item score.

## 3. Results

### 3.1. Participants

**Figure 1:**
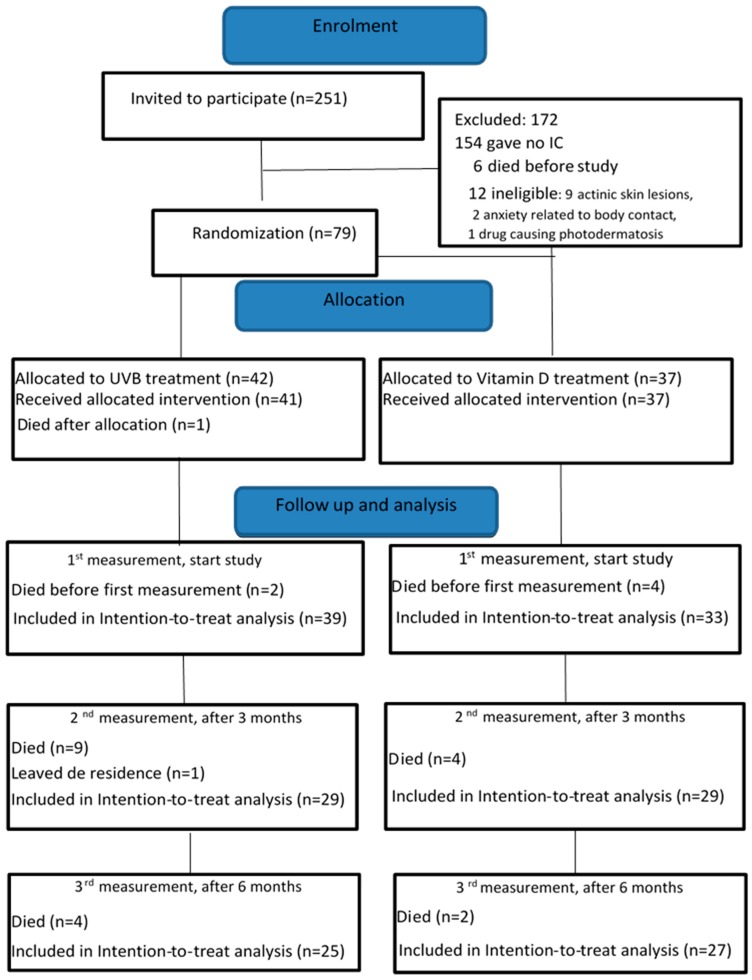
Enrolment illustrated in a CONSORT 2010 flow diagram. IC: informed consent, UVB: ultraviolet B.

This study was carried out between October 2016 and April 2017 in two nursing homes, and between October 2017 and April 2018 in a third nursing home. We started with the trial at the third location later because the number of persons who gave informed consent from the first two locations were not enough to reach the calculated power of the study. The legal representatives of seventy-nine nursing home residents gave informed consent to participate in the study (Figure 1).

Table 1 shows the baseline characteristics of the study participants in the VD and UVB groups. There was no significant difference between the two groups concerning the primary outcome well-being (agitation and depression) or on QoL measures determined using QUALIDEM. Regarding baseline data on biochemical markers of bone homeostasis, 18 measurements were missing (8 in the VD group and 10 in the UVB group) due to logistical problems at the laboratory. Linking laboratory patient’s numbers with trial numbers failed and therefore the source of the samples could not be identified. The baseline serum concentration of 25(OH)D3 in the 78 nursing home residents was significantly lower in the UVB group, with a median of 66.4 (IQR, 53.6–78.7), versus 86.4 (IQR, 65.1–99.7) in the VD group.

### 3.2. Adherence of Nursing Home Residents to the Intervention

Twelve of the participants (30%) in the UVB group refused to adhere to the intervention procedure following initial sessions for a variety of reasons, including an unwillingness to remove clothes or to wear protective glasses, feeling cold or anxious, not understanding the purpose of the procedure or being unable to lie quietly on a bed during UVB exposure. The other participants (70%) showed variable adherence to the UVB treatment regime or died before the end of the treatment period, which resulted in the following duration of the UVB exposure: 8 of the participants (19%) completed UVB sessions of between 24 and 100 min in total, 3 (7%) between 100 and 200, 14 (34%) between 200 and 300 min and 4 (10%) between 300 and 400 min (when participants clearly objected to the UVB session it was discontinued). Eleven (28%) of the participants experienced the sessions as being pleasant and reinforcing, as observed by the nursing staff.

### 3.3. Effect of UVB on the Outcome Variables

Table 2 shows the results of multilevel analyses of effects on the primary and secondary outcomes.

#### 3.3.1. Effect of UVB Treatment on Well-Being

No significant between-group differences were observed for the primary outcome measures. With the VD group as a reference, the CMAI estimated difference between mean change scores was 4.4 (95% CI −2.3 to 11.2, *p* = 0.194) at three months and −0.2 (95% CI −6.8 to 7.2, *p* = 0.953) at six months. The Cornell estimated difference was 1.3 (95% CI −1.9 to 4.6, *p* = 0.412) at three months and −1.3 (95% CI −4.5 to 1.9, *p* = 0.427) at six months.

#### 3.3.2. Effect of UVB Treatment on Secondary Outcomes

Quality of life as measured by QUALIDEM showed a significant difference between groups and over time on the subscale “restless/tense behavior”. With the VD group as a reference, the estimated difference between mean change scores on restless/tense behavior was −1.1 (95% CI −2.1 to −0.1, *p* = 0.025) at three months and 1.1 (95% CI 0.1 to 2.1, *p* = 0.042) at six months. The linear mixed model analysis showed a significant time x group interaction effect (*p* = 0.003), with less restless/tense behavior at six months in the UVB group with the VD group as a reference, compared to the three months outcomes (estimated difference between mean change scores 2.2, 95% CI 0.8 to 3.6).

The 25(OH)D3 serum concentrations in the UVB group at six months was lower in comparison to the VD group, with an estimated difference between mean scores of −9.3 (95% CI −19.4 to 1.0, *p* = 0.073) at three months and -21.9 (95% CI −32.6 to −11.2, *p* < 0.001) at six months.

No significant between-group differences were observed for the remaining biochemical parameters of bone homeostasis (data not shown).

#### 3.3.3. Harmful or Adverse Events

Transitional redness of the skin was observed in 3 participants in the UVB group, although this disappeared after 24 h.

### 3.4. Additional (Sensitivity) Analysis

Because of the variability in duration of UVB exposure in the intervention group, we performed an additional analysis, keeping those who maintained any duration of UVB exposure as “UVB-exposed” and moving those who refused the intervention to the control group (12 participants) [Appendix A Table A1]. No significant between-group difference was observed for the primary outcome measures. Quality of life as measured by QUALIDEM showed a difference between groups and over time on the subscale “restless/tense behavior”, *p* = 0.012. With the VD group as a reference, the estimated difference between mean change scores on restless/tense behavior was −0.6 (95% CI −1.7 to 0.4, *p* = 0.207) at three months and 1.2 (95% CI 0.2 to 2.3, *p* = 0.025) at six months.

## 4. Discussion

In this study, the first randomized control trial to assess the effect of UVB on agitation and depression in people with dementia, we found no significant effect of UVB light on the well-being of nursing home residents. By comparison, in a population of dermatological patients and healthy volunteers, Edstrom et al. reported a significant improvement in scores on the Montgomery Asberg Depression Rating Scale (MADRS) after six weeks (2–3 sessions weekly) of whole body UVB exposure [3]. The difference in results may be attributable to lower treatment adherence and a smaller body area exposed to UVB light amongst our participants.

Our study showed an increase in restless/tense behavior in the UVB group in the first three months and less restless/tense behavior in the same group in the second three-month period compared to the control group. The additional analysis showed no difference between two groups in the first three months and the same results in the second three months. A similar effect was found in a study by Gambichler et al., where healthy volunteers reported feeling more balanced and less nervous after three weekly sessions of whole-body UVA exposure [26]. A positive effect of UVB light on restless/tense behavior in this study population was observed after six months. This could be due to an adaptation period in which participants with advanced dementia became accustomed to a change in their daily routine, late response to the treatment or dose response to the treatment. To look at the normal progression of the restless behavior in people with dementia in nursing homes, we referred to the study of Mjorud et al., a 10 months follow up of persons with dementia living in nursing homes [27]. The authors observed that 19.6% of the participants improved in the course of 10 months on the tension scale of the QUALID, 35.7% remained stable and 44.5% worsened. This variance of 34.6% was associated with changes in the clinical dementia rating, NPI scores and baseline tension score. The mechanisms that can be triggered by UV light to modulate positive psychological effects are: (A) through the vitamin D receptors in the brain [28,29] and (B) through the skin affecting three local systems: (i) the skin analog of the hypothalamic-pituitary-adrenal (HPA) axis [30], (ii) the serotoninergic/melatoninergic system [31] and (iii) the immune system [32]. The effect of UV light exerted through skin is a process assumed to interplay with systemic mechanisms of body homeostasis, involving the paraventricular and arcuate nuclei of hypothalamus and triggering rapid stimulation of the brain [33].

Serum concentrations of 25(OH)D3 increased significantly in the VD group in the last three month period in comparison to the UVB group. This was not in line with the pilot study of Chel et al. which showed a significant increase in 25(OH)D3 in persons with dementia after 8 weeks of UVB exposure and this was not the case in our study. This could be due to differences in adherence to the prescribed regime, an inability of older skin to synthesize 25(OH)D3 over a longer period or to 25(OH)D3 reaching a plateau (81.5% of our participants in the UVB group were VD sufficient (25(OH)D >50 nmol/L [34]) in comparison with the pilot study where the participants were VD deficient or insufficient [35,36].

A major strength of our study was the multicenter RCT design, which included a six-month follow-up period. We also carried out an intention-to-treat analysis that provides not only an estimation of the effect of treatment but also the applicability of the procedure in this specific population.

The main limitations of our study were the lack of blinding and the low adherence to the intervention by nursing home residents with dementia. To partially reduce the last limitation, we performed a sensitivity analysis comparing the participants who actually were UVB exposed with participants who were not UVB exposed. The additional analysis showed the same results as the intention-to-treat analysis for the main outcomes of the study.

There are strategic lessons to be learned from this study, especially for researchers dedicated to the population of people with dementia. In terms of adherence, it was really difficult to have all the participants stick with the intervention. For ethical reasons, UVB treatment was discontinued when participants clearly objected or showed signs of discomfort on two consecutive sessions. The use of sunbeds by nursing home residents with dementia also highlighted certain practical problems underlying the low adherence, including feeling cold, anxious, being unable to lie still or being unable to understand the purpose of the procedure. Future research efforts in this field should first attempt to find more comfortable approaches to administering UVB light.

The effect of UVB light on wellbeing has not yet been examined in this population. In our study, the amount of the UVB light administered was calculated on the base of the UVB light needed to sustain a sufficient 25(OH)D3 serum concentration. The exposure needed to achieve any effect on agitation, depression or quality of life is not yet known. It is possible that a better adherence to the prescribed regime or a more intensive treatment than the treatment our participants actually received might present other results on the effect of UVB on wellbeing and quality of life.

## 5. Conclusions

The exposure of nursing home residents with dementia to UVB light showed no positive benefits in terms of wellbeing. UVB treatment may have a positive effect on the restless/tense behavior characteristic of advanced dementia, but more research is needed to confirm this finding.

## Figures and Tables

**Table 1:** Characteristics of the participants at baseline by study group.

Variable	UVB	Vitamin D	*p*-Value
**Gender %,(n)**			0.20 ^a^
male	24 (10)	38 (14)	
Female	76 (31)	62 (23)	
**Age in years, mean (SD)**	84.2 (79.5–87.5)	83.6 (77.5–88.5)	0.74 ^b^
Fitzpatrick skin scale %,(n)			0.90 ^c^
1. always burns easily, never tans	0	3 (1)	
2. always burns easily, tans slightly	66 (27)	62 (23)	
3. burns moderately, tans gradually	30 (12)	32 (12)	
4. burns minimally, tans moderately	0	0	
5. rarely burns, tans profusely	5 (2)	0	
6. never burns, tans profusely	0	3 (1)	
**Dementia severity, mean BANS-S (SD)**	15.1 (4.3)	16.6 (5.7)	0.20 ^b^
**Agitation (median CMAI, IQR)**	40.0 (30.3–62.5)	41.0 (30.5–61.0)	0.82 ^d^
**Cornell Scale For Depression (Median, IQR)**	9.5 (4.9–13.0)	9.5 (5.0–12.0)	0.88 ^d^
**QUALIDEM (Median, IQR)**			
A. Care relationship	6.0 (4.0–8.0)	7.0 (5.0–8.5)	0.28 ^d^
B. Positive affect	10.0 (7.5–12.0)	9.0 (8.0–11.5)	0.63
C. Negative affect	3.0 (3.0–4.0)	4.0 (3.0–4.0)	0.98
D. Restless/tense behavior	6.0 (2.5–8.0)	6.0 (3.0–8.0)	0.91
E. Social relations	6.0 (4.5–8.0)	6.0 (4.0–7.0)	0.45
G. Social isolation	6.5 (4.0–9.0)	8.0 (5.0–9.0)	0.52
**Blood tests (Median, IQR)) ^#^**			
Creatinine (µmol/L)	73.0 (61.0–982.0)	72.5 (56.0–988.2)	0.82 ^d^
Calcium (mmol/L)	2.3 (2.3–92.5)	2.3 (2.3–92.4)	0.81
Phosphate (mmol/L)	1.1 (1.0–91.1)	1.1 (0.9–91.3)	0.60
Alkaline phosphatase (U/I)	86.4 (65.3–9116.6)	82.4 (70.0–997.9)	0.96
25(OH)D3 (nmo/L)	66.4 (53.6–978.7)	86.4 (65.1–999.7)	0.04
Parathyroid hormone (pmol/L)	5.7 (3.0–97.6)	3.5 (2.7–96.5)	0.24

^a^ Pearson’s Chi-squared test used for gender; ^b^ Students t-test for age and BANS-S; ^c^ Kruskal-Wallis test for skin type; ^d^ Mann-Whitney test for the other parameters. ^#^ Missing: Vitamin D, *n* = 11; UVB, *n* = 14, IQR—Interquartile range, CMAI—Cohen-Mansfield Agitation Inventory, BANS-S—Bedford Alzheimer Nursing Severity Scale, 25(OH)D3: serum 25-hydroxyvitamin D.

**Table 2:** Estimated marginal group means and *p*-values, based on mixed model analysis *.

	3 Months (*n* = 58)			6 Months (*n* = 52)			*p*-Value		
	Estimated Mean	Adjusted MD	*p*-Value	Estimated Mean	Adjusted MD	*p*-Value	Pg	Pt	Pgt
	Score (95% CI)	(95% CI)		Score (95% CI)	(95% CI)				
**CMAI total score**							0.431	0.076	0.258
**UVB**	49.4 (44.7, 54.0)	4.4 (−2.3, 11.2)	0.194	50.6 (45.7, 55.5)	−0.2 (−6.8, 7.2)	0.953			
**VD**	45.0 (41.0, 49.8.2)			50.4 (45.4, 55.5)					
**Cornell scale for depression**							0.982	0.014	0.246
**UVB**	8.5 (6.4, 10.7)	1.3 (−1.9, 4.6)	0.412	10.1 (8.0, 12.2)	−1.3 (−4.5, 1.9,)	0.427			
**VD**	7.2 (4.8, 9.7)			11.4 (9.0, 13.8)					
**Care relationship (QUAL)**							0.776	0.421	0.307
**UVB**	6.2 (5.8, 7.0)	−0.2 (−1.0, 0.6)	0.684	6.3 (5.7, 6.7)	0.3 (−0.5, 1.2)	0.402			
**VD**	6.4 (5.4, 6.5)			6.3 (5.7, 6.7)					
**Positive affect (QUAL.)**							0.698	0.363	0.646
**UVB**	8.8 (7.9, 9.7)	0.4 (−0.9, 1.6)	0.555	8.9 (8.0, 9.9)	0.0 (−1.3, 1.3)	0.947			
**VD**	8.4 (7.5, 9.3)			8.9 (8.0, 9.8)					
**Negative affect (QUAL.)**							0.303	0.507	0.866
**UVB**	3.4 (3.0, 3.7)	−0.2 (−0.7, 0.3)	0.483	3.3 (3.0, 3.7)	−0.2 (−0.7, 0.3)	0.377			
**VD**	3.6 (3.2, 3.9)			3.5 (3.1, 3.8)					
**Restless/Tense (QUAL.)**							0.937	0.520	0.003
**UVB**	4.6 (3.9, 5.1)	−1.1 (−2.1, −0.1,)	0.025	5.5 (4.8, 6.2)	1.1 (0.1, 2.1,)	0.042			
**VD**	5.7 (5.0, 6.4)			4.4 (3.7, 5.1)					
**Social relations (QUAL.)**							0.960	0.920	0.763
**UVB**	5.7 (5.1, 6.3)	−0.1 (−1.0, 0.8)	0.813	5.8 (5.1, 6.5)	0.1 (−0.8, 1.0)	0.879			
**VD**	5.8 (5.1, 6.4)			5.7 (5.1, 6.4)					
**Social isolation (QUAL.)**							0.329	0.441	0.158
**UVB**	5.9 (5.3, 6.6)	−0.8 (−1.7, 0.2)	0.104	6.2 (5.5,6.8)	0.0 (−1.0, 1.0)	0.988			
**VD**	6.7 (6.1, 7.4)			6.2 (5.5, 6.8)					
**25(OH)D3**							0.003	0.141	0.001
**UVB**	66.3 (59.1, 73.5)	−9.3 (−19.4, 1.0)	0.073	62.7 (54.9, 70.5)	−21.9 (−32.6, −11.2)	0.000			
**VD**	75.6 (69.0, 82.1)			84.6 (77.9, 91.3)					

CMAI (Cohen-Mansfield agitation inventory): higher scores indicate a higher level of agitation; Cornell scale for depression: higher scores indicate more depressive symptoms; QUALIDEM: higher scores indicate higher QoL; 25(OH)D3: serum 25-hydroxyvitamin D3; * The mixed model analysis adjusted for the baseline of the outcome measures shows the *p*-values for the intervention (UVB) versus control (VD) condition (Pg), the overall time effect (Pt) and the interaction effect of group and time (Pgt). The treatment effect is presented as adjusted mean difference (MD) between the VD and UVB groups for each time point with the VD group as a reference category.

## Appendix A

**Table A1:** Estimated marginal group means and p-values, based on mixed model analysis, additional analysis *.

	3 Months (*n* = 58)			6 Months (*n* = 52)			*p*-Value		
	Estimated Mean	Adjusted MD	*p*-Value	Estimated Mean	Adjusted MD	*p*-Value	Pg	Pt	Pgt
	Score (95% CI)	(95% CI)		Score (95% CI)	(95% CI)				
**CMAI total score**							0.847	0.175	0.155
**UVB**	49.3 (43.9, 54.7)	3.3 (−3.6, 10.2)	0.343	49.2 (43.5–54.8)	−2.2 (−9.4, 5.0)	0.554			
**VD**	46.0 (41.7, 50.3)			51.3 (46.7–55.8)					
**Cornell scale for depression**							0.483	0.032	0.260
**UVB**	8.5 (6.4, 10.7)	2.1 (−1.9, 4.6)	0.200	10.1 (8.0–12.2)	−0.5 (−2.7, 3.6)	0.775			
**VD**	7.1 (5.0, 9.1)			11.0 (8.8, 12.9)					
**Care relationship (QUAL)**							0.575	0.617	0.285
**UVB**	6.0 (5.4, 6.7)	−0.5 (− 1.3, 1.3)	0.259	6.2 (5.5, 6.8)	0.3 (−0.5, 1.2)	0.820			
**VD**	6.5 (6.0, 7.0)			6.1 (5.6, 6.7)					
**Positive affect (QUAL.)**							0.827	0.602	0.171
**UVB**	8.8 (7.8, 9.9)	0.4 (−0.9, 1.6)	0.561	8.5 (7.5, 9.6)	0.0 (−1.3, 1.3)	0.354			
**VD**	8.5 (7.7, 9.3)			9.1 (8.3, 9.9)					
**Negative affect (QUAL.)**							0.945	0.337	0.218
**UVB**	3.6 (3.2, 4.0)	0.2 (−0.3, 0.7)	0.452	3.3 (2.8,3.7)	−0.2 (−0.7, 0.3)	0.406			
**VD**	3.4 (3.1, 3.7)			3.5 (3.2, 3.8)					
**Restless/Tense (QUAL.)**							0.969	0.462	0.012
**UVB**	4.8 (4.0, 5.6)	−0.6 (−1.7, 0.4,)	0.207	5.7 (4.8, 6.5)	1.2 (0.2, 2.3,)	0.025			
**VD**	5.4 (4.8, 6.0)			4.5 (3.7, 5.1)					
**Social relations (QUAL.)**							0.939	0.857	0.324
**UVB**	5.9 (5.2, 6.7)	0.3 (−0.6, 1.2)	0.484	5.6 (4.9, 6.4)	−0.3 (−1.2, 0.7)	0.573			
**VD**	5.6 (5.1, 6.2)			5.9 (5.3, 6.5)					
**Social isolation (QUAL.)**							0.292	0.557	0.546
**UVB**	6.0 (5.2, 6.8)	−0.6 (−1.6, 0.4)	0.223	6.0 (5.2, 6.8)	−0.3 (−1.3, 0.7)	0.596			
**VD**	6.6 (6.0, 7.2)			6.2 (5.6, 6.7)					
**25(OH)D3**							0.039	0.237	0.005
**UVB**	67.6 (59.1, 76.0)	−5.1 (−15.8, 5.6)	0.344	64.1 (54.9, 73.3)	−16.8 (−28.0, −5.5)	0.004			
**VD**	72.7 (66.5, 78.9)			80.7 (74.5, 87.1)					

CMAI (Cohen-Mansfield agitation inventory): higher scores indicate a higher level of agitation; Cornell scale for depression: higher scores indicate more depressive symptoms; QUALIDEM: higher scores indicate higher QoL; 25(OH)D3: serum 25-hydroxyvitamin D3. * The mixed model analysis adjusted for the baseline of the outcome measures shows the *p*-values for the intervention (UVB) versus control (VD) condition (Pg), the overall time effect (Pt) and the interaction effect of group and time (Pgt). The treatment effect is presented as adjusted mean difference (MD) between the VD and UVB group for each time point with VD group as reference category.

## References

[1] Lindqvist P.G., Epstein E., Nielsen K., Landin-Olsson M., Ingvar C., Olsson H. (2016). Avoidance of sun exposure as a risk factor for major causes of death: A competing risk analysis of the Melanoma in Southern Sweden cohort. J. Intern. Med..

[2] Holick M.F. (2005). Vitamin D for health and in chronic kidney disease. Semin. Dial..

[3] Edstrom D.W., Linder J., Wennersten G., Brismar K., Ros A.M. (2010). Phototherapy with ultraviolet radiation: A study of hormone parameters and psychological effects. J. Eur. Acad. Dermatol. Venereol..

[4] Pudikov I.V., Dorokhov V.B. (2012). The special physiological importance of the UV-A spectrum for successful phototherapy. Hum. Physiol..

[5] Taylor S.L., Kaur M., LoSicco K., Willard J., Camacho F., O’Rourke K.S., Feldman S.R. (2009). Pilot study of the effect of ultraviolet light on pain and mood in fibromyalgia syndrome. J. Altern. Complement. Med..

[6] Gambichler T., Bader A., Vojvodic M., Avermaete A., Schenk M., Altmeyer P., Hoffmann K. (2002). Plasma levels of opioid peptides after sunbed exposures. Br. J. Dermatol..

[7] Knippenberg S., Damoiseaux J., Bol Y., Hupperts R., Taylor B.V., Ponsonby A.L., Dwyer T., Simpson S., van der Mei I.A. (2014). Higher levels of reported sun exposure, and not vitamin D status, are associated with less depressive symptoms and fatigue in multiple sclerosis. Acta Neurol. Scand..

[8] Meffert H., Scherf H.-P., Piazena H. (2006). Systemic effects of infrared and ultraviolet irradiations—A prospective, placebo-controlled intra-individual comparison. German Systemische wirkungen infraroter und ultravioletter strahlen: Ein prospektiver, plazebokontrollierter, intraindividueller vergleich. Aktuelle Dermatol..

[9] Veleva B.I., van Bezooijen R.L., Chel V.G.M., Numans M.E., Caljouw M.A.A. (2018). Effect of ultraviolet light on mood, depressive disorders and well-being. Photodermatol. Photoimmunol. Photomed..

[10] Holick M.F. (2016). Biological Effects of Sunlight, Ultraviolet Radiation, Visible Light, Infrared Radiation and Vitamin D for Health. Anticancer Res..

[11] Chel V.G., Ooms M.E., Pavel S., de Gruijl F., Brand A., Lips P. (2011). Prevention and treatment of vitamin D deficiency in Dutch psychogeriatric nursing home residents by weekly half-body UVB exposure after showering: A pilot study. Age Ageing.

[12] Broe K.E., Chen T.C., Weinberg J., Bischoff-Ferrari H.A., Holick M.F., Kiel D.P. (2007). A higher dose of vitamin d reduces the risk of falls in nursing home residents: A randomized, multiple-dose study. J. Am. Geriatr. Soc..

[13] Juzeniene A., Moan J. (2012). Beneficial effects of UV radiation other than via vitamin D production. Dermatoendocrinol.

[14] Chenoweth L., King M.T., Jeon Y.H., Brodaty H., Stein-Parbury J., Norman R., Haas M., Luscombe G. (2009). Caring for Aged Dementia Care Resident Study (CADRES) of person-centred care, dementia-care mapping, and usual care in dementia: A cluster-randomised trial. Lancet Neurol..

[15] Morley J.E., Vellas B., van Kan G.A., Anker S.D., Bauer J.M., Bernabei R., Cesari M., Chumlea W.C., Doehner W., Evans J. (2013). Frailty consensus: A call to action. J. Am. Med. Dir. Assoc..

[16] Klapwijk M.S., Caljouw M.A., Pieper M.J., van der Steen J.T., Achterberg W.P. (2016). Characteristics Associated with Quality of Life in Long-Term Care Residents with Dementia: A Cross-Sectional Study. Dement. Geriatr. Cogn. Disord..

[17] Steptoe A., Deaton A., Stone A.A. (2015). Subjective wellbeing, health, and ageing. Lancet.

[18] Hendriks S.A., Smalbrugge M., Galindo-Garre F., Hertogh C.M., van der Steen J.T. (2015). From admission to death: Prevalence and course of pain, agitation, and shortness of breath, and treatment of these symptoms in nursing home residents with dementia. J. Am. Med. Dir. Assoc..

[19] He S.Y., McCulloch C.E., Boscardin W.J., Chren M.M., Linos E., Arron S.T. (2014). Self-reported pigmentary phenotypes and race are significant but incomplete predictors of Fitzpatrick skin phototype in an ethnically diverse population. J. Am. Acad. Dermatol..

[20] (2012). Evaluation of Dietary Reference Values for Vitamin D.

[21] Cohen-Mansfield J., Marx M.S., Rosenthal A.S. (1989). A description of agitation in a nursing home. J. Gerontol..

[22] Miller R.J., Snowdon J., Vaughan R. (1995). The use of the Cohen-Mansfield Agitation Inventory in the assessment of behavioral disorders in nursing homes. J. Am. Geriatr. Soc..

[23] Alexopoulos G.S., Abrams R.C., Young R.C., Shamoian C.A. (1988). Cornell Scale for Depression in Dementia. Biol. Psychiatry.

[24] Ettema T.P., Droes R.M., de Lange J., Mellenbergh G.J., Ribbe M.W. (2007). QUALIDEM: Development and evaluation of a dementia specific quality of life instrument. Scalability, reliability and internal structure. Int. J. Geriatr. Psychiatry.

[25] West B.T. (2009). Analyzing longitudinal data with the linear mixed models procedure in SPSS. Eval. Health Prof..

[26] Gambichler T., Bader A., Vojvodic M., Bechara F.G., Sauermann K., Altmeyer P., Hoffmann K. (2002). Impact of UVA exposure on psychological parameters and circulating serotonin and melatonin. BMC Dermatol..

[27] Mjorud M., Rosvik J., Rokstad A.M., Kirkevold M., Engedal K. (2014). Variables associated with change in quality of life among persons with dementia in nursing homes: A 10 months follow-up study. PLoS ONE.

[28] Alpert J.S. (2010). Sunshine: Clinical friend or foe?. Am. J. Med..

[29] Cui X., Gooch H., Petty A., McGrath J.J., Eyles D. (2017). Vitamin D and the brain: Genomic and non-genomic actions. Mol. Cell. Endocrinol..

[30] Slominski A., Wortsman J. (2000). Neuroendocrinology of the skin. Endocr. Rev..

[31] Slominski A., Wortsman J., Tobin D.J. (2005). The cutaneous serotoninergic/melatoninergic system: Securing a place under the sun. FASEB J..

[32] Skobowiat C., Postlethwaite A.E., Slominski A.T. (2017). Skin Exposure to Ultraviolet B Rapidly Activates Systemic Neuroendocrine and Immunosuppressive Responses. Photochem. Photobiol..

[33] Slominski A.T., Zmijewski M.A., Plonka P.M., Szaflarski J.P., Paus R. (2018). How UV Light Touches the Brain and Endocrine System Through Skin, and Why. Endocrinology.

[34] Holick M.F., Binkley N.C., Bischoff-Ferrari H.A., Gordon C.M., Hanley D.A., Heaney R.P., Murad M.H., Weaver C.M., Endocrine S. (2011). Evaluation, treatment, and prevention of vitamin D deficiency: An Endocrine Society clinical practice guideline. J. Clin. Endocrinol. Metab..

[35] Holick M.F. (2017). Ultraviolet B Radiation: The Vitamin D Connection. Adv. Exp. Med. Biol..

[36] Durvasula S., Gies P., Mason R.S., Chen J.S., Henderson S., Seibel M.J., Sambrook P.N., March L.M., Lord S.R., Kok C. (2014). Vitamin D response of older people in residential aged care to sunlight-derived ultraviolet radiation. Arch. Osteoporos..

